# Description and removal of background activity in EEG power spectra under general anesthesia using the Lorentzian curve

**DOI:** 10.1186/1471-2202-16-S1-P233

**Published:** 2015-12-18

**Authors:** Mariia Fedotenkova, Axel Hutt, James W Sleigh

**Affiliations:** 1 CNRS, Loria, UMR nº 7503, Vandœuvre-lès-Nancy, F-54500, France; 2 NEUROSYS team, Inria, Villers-lès-Nancy, F-54600, France; 3 Université de Lorraine, Loria, UMR nº 7503, Vandœuvre-lès-Nancy, France; 4 Department of Anesthesia, Waikato Clinical School of the University of Auckland, Waikato Hospital, Hamilton 3206, New Zealand

## 

General anesthesia is an important medical procedure in today's hospital practice and comprises loss of consciousness, analgesia, amnesia and immobility. Our current work analyzes patient reaction on nociception stimuli during a surgical operation and differences in this reaction provided different anesthetic drugs, propofol and desflurane. The studied dataset comprises EEG-recordings before and after incision obtained from 115 patients [1]. The task is the identification of spectral EEG signal features reflecting the incision. This analysis will reveal a possible new marker of pain during deep anesthesia.

This work considers one of the approaches to the problem, namely spectral analysis. First, power spectral density (PSD) estimates were obtained using Welch's method. It is well known that EEG power spectrum decays with higher frequencies following *~1/f *scaling [2-4]. We attribute this behavior to background activity [5], which takes place in the brain when no other activity is present. Background activity was describe by fitting regression curve *P(f)~a/f ^b ^* to each PSD estimate. However, the resulting goodness of fit was not satisfactory. It is due to rise of power in delta peak, which becomes prominent under general anesthesia and makes the process of curve fitting less reliable. Thus, the original model was substituted by the Lorentzian function *P(f)=a/(f ^b ^+ c)*, which resembles the shape of actual power spectrum quite well. Afterwards, regression curves were subtracted from each power spectrum to normalize it [3] and to analyze spectral power contained in delta and alpha peaks regardless of distinctions in patients.

The results of this work revealed small differences between propofol and desflurane. Power spectra of patients receiving desflurane have more regular shape than the ones from propofol group. It can also be seen that delta power remains more consistent, while alpha power varies greatly from patient to patient. Another result of this work is a trend in the distribution of Lorentzian curve parameters: the set of parameters remains compact for small values of *b*, but *a *and *c *scatters significantly when *b *(which corresponds to steepness of curve) is larger than three. Results of this work provide insights on underlying background activity. However, they do not allow to distinguish between pre- and post-incision and poorly between propofol and desflurane. This problem requires more complicated techniques. Future work will expand spectral analysis with time information (time-frequency representations) and investigate time structure by means of recurrence analysis.

**Figure 1:**
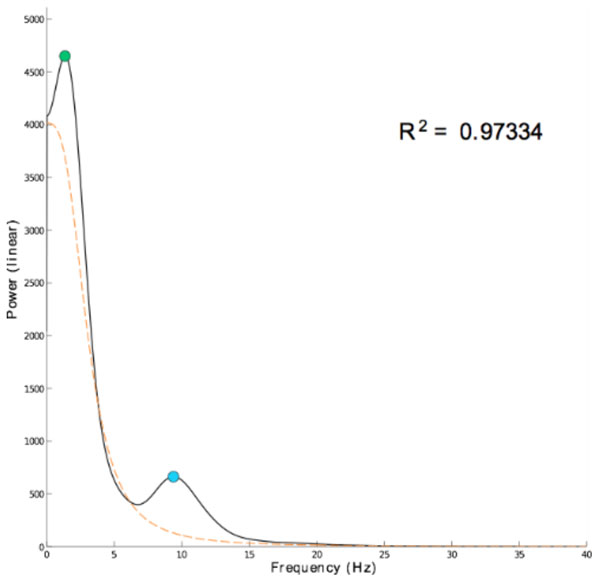
**Example of power spectrum density estimate (solid black), with fitted Lorentzian curve (dashed orange), goodness of fit (R2), delta (green circle) and alpha peaks (blue circle)**.
